# Asymptomatic Cryptosporidiosis in Children Living with HIV

**DOI:** 10.3390/tropicalmed7110352

**Published:** 2022-11-04

**Authors:** Ajib Diptyanusa, Ika Puspa Sari, Agnes Kurniawan

**Affiliations:** 1 Center for Tropical Medicine, Faculty of Medicine, Public Health and Nursing, Universitas Gadjah Mada, Yogyakarta 55281, Indonesia; 2 Study Program of Medical Specialist in Clinical Parasitology, Faculty of Medicine, Universitas Indonesia, Jakarta 10430, Indonesia; 3 Department of Parasitology, Faculty of Medicine, Universitas Indonesia, Jakarta 10430, Indonesia

**Keywords:** children, opportunistic infection, HIV, drinking water

## Abstract

Children living with human immunodeficiency virus (HIV) have an increased risk of opportunistic *Cryptosporidium* infection. *Cryptosporidium* usually causes chronic diarrhea that may lead to impaired growth and cognitive function in children. This study aimed to estimate the prevalence of cryptosporidiosis in children, describe its clinical characteristics, and the risk factors. A cross-sectional study involving children aged 6 months to 18 years old with confirmed HIV infection was carried out in Sardjito General Hospital, Yogyakarta. Diagnosis of cryptosporidiosis was made by PCR of 18S rRNA after being screened by microscopic examination. The clinical characteristics and risk factors were obtained from medical records and structured questionnaires. A total of 52 participants were included in the final analysis. The prevalence of cryptosporidiosis was 42.3%. Approximately 68% of the HIV children with cryptosporidiosis were asymptomatic, while those who reported symptoms showed weight loss and diarrhea. Independent risk factors of cryptosporidiosis were diarrhea (AOR 6.5; 95% CI 1.16–36.67), well water as drinking water source (AOR 6.7; 95% CI 1.83–24.93), and drink untreated water (AOR 5.8; 95% CI 1.04–32.64). A high prevalence of asymptomatic cryptosporidiosis was observed among children with HIV infection and PCR screening of *Cryptosporidium* in high-risk children is advisable.

## 1. Introduction

Individuals living with human immunodeficiency virus (HIV) are generally at risk of getting opportunistic infections, and diarrhea is one of the most common presenting complaints of opportunistic infections in this group [1]. Cryptosporidiosis caused by the coccidian parasite *Cryptosporidium* spp. is one of the most common opportunistic infections causing diarrheal disease in individuals living with HIV [2]. The parasite is known to be transmitted through the fecal–oral route via contaminated food or water [3], and the risk of infection is reported to be higher in persons with a CD4^+^ count of less than 100 cells/mL [4]. In general, the prevalence of human cryptosporidiosis in developing countries was reported to be around 20% [5,6], while in Indonesia the proportion of infection ranged from 2.1% to 60.9% in both symptomatic and asymptomatic individuals [7,8,9]. The incidence of cryptosporidiosis was found to be predominant in individuals living with HIV [10,11]. Globally, the pooled prevalence of human cryptosporidiosis in people living with HIV was estimated to be approximately 14.0%, owing mainly from reported studies in Sub-Saharan Africa and Asia [12].

In many high-burden countries, diagnoses of cryptosporidiosis rely mostly on microscopic identification of the parasites [13], including in our daily practice. However, microscopic identification of the parasite can sometimes be observer-dependent and challenging [14,15]. Additionally, asymptomatic infections may occur and these individuals serve as a source of parasite transmission if left untreated [16]. Data on cryptosporidiosis in children living with HIV in Indonesia are limited [12]. These reasons have led to underdiagnosis and delayed management, causing chronic complications including delayed growth and development, as well as impaired cognitive function [17,18]. Therefore, a study on cryptosporidiosis in HIV-positive children is vital, as the proportion of newly diagnosed children with HIV infection has been increasing over the years [19,20]. The current study aimed to estimate the prevalence, describe clinical characteristics, and identify risk factors of cryptosporidiosis in children with HIV infection.

## 2. Materials and Methods

### 2.1. Study Design

A cross-sectional study was conducted at the pediatric outpatient clinic of Sardjito General Hospital in Yogyakarta, Indonesia, from April through October 2021. The Sardjito General Hospital is a 700-bed, tertiary-level referral hospital at the provincial level. The hospital serves patients of all financial capabilities. HIV-related services are provided in a specialized HIV clinic for adult patients and integrated in the daily polyclinic for pediatric patients.

### 2.2. Study Population

Children aged 6 months through <18 years diagnosed with HIV infection whose parents or guardian gave consent were included in the study. Exclusion criteria were children on antidiarrheal medication, and children under therapies using either antibiotics, anthelminthics, or antiparasitic drugs. Total population sampling method was used in current study, which included 59 pediatric HIV patients in the clinic’s patient registry. All 59 patients were included in the study regardless of the presence or absence of diarrhea or other gastrointestinal symptoms at the moment of evaluation.

### 2.3. Assessment of Risk Factors

A structured questionnaire was used to identify behavioral risks among study subjects. Survey was conducted on the caregiver or mother of the study subjects. The question items included demographic information, exposure to animals or natural water, dietary habits, food hygiene, drinking water source, and defecation practices. Clinical characteristics of study subjects were extracted from the medical records, including current and past medical history, diarrhea, nausea and vomiting, abdominal pain, anorexia, results of vital signs, height and weight measurements, signs of dehydration, lymphadenopathy, organomegaly, abdominal tenderness, extremity edema, CD4^+^ level, viral load, and medication history. Data were recorded on the case record form.

Nutritional status was classified for all subjects using weight/height (age < 5 years) or body mass index (BMI)/age (age ≥ 5 years) according to the WHO criteria: severely thin/wasted, thin/wasted, normal, overweight, and obese [21]. The status of stunting was only defined for subjects aged < 5 years according to the measurement for height/age by the WHO criteria: normal, stunted, and severely stunted [21]. Weight loss was defined when there was a reduction of >5% body weight within the past 1 month. Immunodeficiency status of the study subjects was determined according to CD4^+^ percentage (age < 5 years) or absolute CD4^+^ count (age ≥ 5 years) [22]. Clinical stages of HIV were classified according to the WHO criteria [22].

### 2.4. Specimen Collection

A single fecal specimen with minimum weight of 15 g or 15 mL was collected from study subjects. Fecal specimens were returned to the investigator within 24 h after collection. The specimen was kept inside a cool box with temperature of 2–4 °C during transport to the Laboratory of Parasitology, Faculty of Medicine, Public Health and Nursing Universitas Gadjah Mada. Fecal specimens were divided into two portions: one portion of non-preserved specimen for microscopic identification and one portion for molecular examination using polymerase chain reaction (PCR) was preserved in 100% ethanol and was then kept in freezer (−80 °C). All specimens were examined for *Cryptosporidium* spp. using both microscopic identification and molecular examination.

### 2.5. Microscopic Identification

Microscopic examination using direct and concentration methods were performed as soon as the specimen arrived at the laboratory. Concentration method using standard Ritchie procedure was conducted for all specimens prior to staining [23]; the fecal sediment was then stained using modified Ziehl Neelsen (mZN) technique [24]. A total of 3 stained fecal smears were made from each specimen. Stained fecal smears were microscopically examined by 3 trained, independent observers. Oocysts of *Cryptosporidium* spp. were identified as round oocysts measured 4 to 5.5 µm in diameter and stained pink against a blue background [25]. Wet preparation with lugol and eosin was also performed to detect other intestinal parasites.

### 2.6. Molecular Examination

Molecular examination using nested PCR was conducted on fecal specimens preserved in 100% ethanol. The DNA extraction of *Cryptosporidium* spp. was performed according to manufacturer’s instruction of FavorPrep™ Stool DNA Isolation Mini Kit (Favorgen, Biotech Corp., Ping Tung, Taiwan). Nested PCR was carried out using the following sequence for 35 cycles: denaturation on 98 °C for 45 s, annealing on 55 °C for 45 s, extension on 72 °C for 60 s, and final extension on 72 °C for 7 min. Electrophoresis was carried out on 2% agarose gel with fluorescent staining (SYBR Safe, Invitrogen, Waltham, MA, USA). First nested primers for *Cryptosporidium* spp. 18S rRNA were 5′-TTCTAGAGCTAATACATGCG-3′ and 5′-CCCTAATCCTTCGAAACAGGA-3′ (product of 1325 bp), while second nested primers were 5′-TTCTAGAGCTAATACATGCG-3′ and 5′-CCCATTTCCTTCGAAACAGGA-3′ (product of 826–864 bp) [26].

### 2.7. Data Analysis

Statistical analysis was carried out using SPSS software ver. 25.0 (IBM Corp., Armonk, NY, USA). Univariate analysis was performed using chi-square or Fisher’s exact test for categorical variables and *t*-test or Mann–Whitney U test for continuous variables, whenever appropriate. Multivariate analysis was conducted using logistic regression method (forward stepwise) on variables with a *p*-value of <0.10 to identify risk factors of cryptosporidiosis in current study. Results were shown as an adjusted odds ratio (AOR) with a 95% confidence interval (CI). All variables were considered statistically significant if *p* < 0.05 (two-sided).

## 3. Results

**Figure 1:**
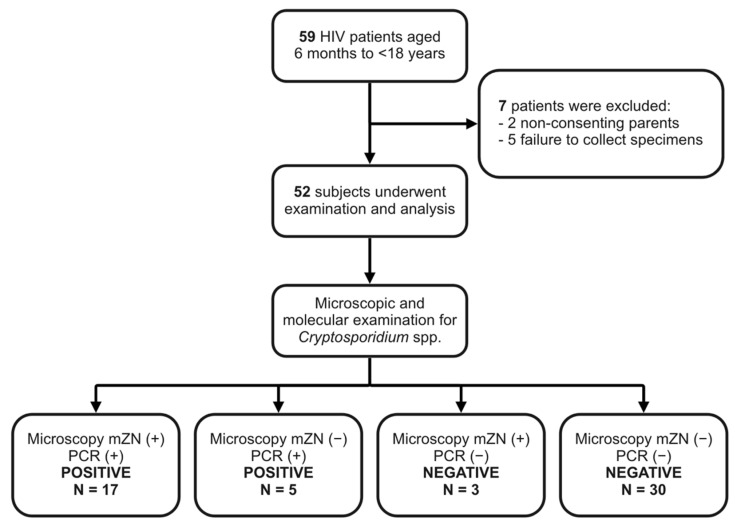
Study flow.

During the study period, a total of 59 pediatric patients with HIV infection were registered. Among these, seven patients were excluded from the study due to non-consenting parents (two patients) and failure to collect fecal specimens (five patients). There were no newly diagnosed HIV patients registered in the clinic during the study period, and the total study population remained 59 subjects. Fifty-two patients were included in the research and in the final analysis (Figure 1).

According to PCR examination, a total of 22 subjects were infected with *Cryptosporidium* spp., resulting in a prevalence of 42.3%. Three subjects who were found positive only by microscopic examination, were then categorized as negative results. Approximately 86% of patients positive for cryptosporidiosis belonged to mild–moderate infection (1–10 oocysts/ microscopic field, 1000× magnification). Other parasites found in the study subjects included *Endolimax nana* (15/52; 28.8%), *Blastocystis* spp. (11/52; 21.1%), *Entamoeba coli* (1/52; 1.9%), and *Chilomastix mesnili* (1/52; 1.9%). A total of 13 subjects with cryptosporidiosis were found to be co-infected with *Blastocystis* spp.

The age of study subjects ranged from 6 months to 16 years, with a female to male ratio of 1:1.4. *Cryptosporidium* was most frequently found in children under 12 years old (*p* > 0.05). Approximately 32.7% subjects had either current, or a history of tuberculosis within the past 1 year, whereas recurrent respiratory infections were found in 11.5% of the enrolled subjects. Median viral load of subjects with cryptosporidiosis was 1,130 copies/mL, higher than those negative for *Cryptosporidium* (400 copies/mL; *p* = 0.204). A total of 21 subjects had an undetectable viral load, among which 6 subjects were found positive for cryptosporidiosis. Approximately 25% (13/52) of subjects suffered from severe immunodeficiency with CD4^+^ count 15% or <200 cells/μL, all of them aged > 5 years. Only two subjects had CD4^+^ < 100 cells/μL, both of which were found positive for cryptosporidiosis. Cryptosporidiosis was more commonly found in subjects with CD4^+^ < 200 cells/μL and who had a higher viral load (*p* > 0.05). The untreated subjects belonged to the children whose parents refused to start the antiretroviral therapy (ART) immediately, or those who were diagnosed around the COVID-19 travel restriction and have not yet received ART until the study period ended. Of the six subjects in the treatment-naïve group, all of them were children under five years, four of which were found undernourished and presented with diarrhea upon visit. General characteristics and clinical findings of the study subjects are described in Table 1.

In this study, the most frequent symptoms reported was weight loss, followed by diarrhea, anorexia, nausea, and vomiting (Table 1). Among subjects who experienced weight loss, 11 subjects belonged to the moderate weight loss group (5–10% loss from initial weight) and 8 subjects suffered from the severe weight loss (loss of >10% of initial weight). All subjects who reported decreased appetite also experienced weight loss. Among 11 subjects who experienced diarrhea, 4 subjects had acute diarrhea (<7 days), 6 subjects with persistent diarrhea (7–14 days), and 1 subject suffered from chronic diarrhea (>14 days). Four subjects with cryptosporidiosis reported recurrent episodes of diarrhea within the past 1 to 2 months. Signs of dehydration such as sunken eyes, dry lips, or decrease skin turgor were also found on physical examination of all subjects who experienced diarrhea. None of the study subjects presented with lymphadenopathy, hepatomegaly or splenomegaly, abdominal tenderness, or extremity edema. The presence of diarrhea and weight loss demonstrated a statistically significant association with cryptosporidiosis in this study. Interestingly, 32% of subjects with cryptosporidiosis did not report symptoms such as diarrhea, fever, weight loss, abdominal pain, nausea, vomiting, bloating, and anorexia.

Table 2 shows more detailed characteristics of children living with HIV with cryptosporidiosis in relation to their HIV stages. Sixteen subjects with cryptosporidiosis were under ART where five subjects received only two nucleoside reverse transcriptase inhibitors (NRTIs), eight subjects received a combination of two NRTIs and one non-nucleoside reverse transcriptase inhibitors (NNRTI), three subjects received combination of two NRTIs and one protease inhibitor. Those who were at stage 3 and 4 HIV received a combination of NRTI and protease inhibitor and were also suffering from moderate or severe cryptosporidiosis, as shown by high number of oocysts on microscopic examination.

Assessment of the risk factors (Table 3) showed that subjects who had close contact with other diarrheic individuals (either parents, siblings, or caregiver), exposure to river water (either domestic or recreational activity), and exposure to animals which lived in close proximity (<10 m) did not show any significant difference in contracting *Cryptosporidium* infection. The same finding was also found with raw vegetable consumption behavior and hand washing practice prior to eating (*p* > 0.05). However, a statistically significant difference was observed among subjects using well water as a drinking water source. Higher incidence of cryptosporidiosis was found in this group compared to those drinking rain/bottled water and no cryptosporidiosis cases were observed in the group utilizing treated/tap water as their drinking water source. The risk behavior of cryptosporidiosis-positive subjects in contrast to drinking water treatment can be found in the Supplementary File (Table S1).

Multivariate analysis was performed on selected variables found to be statistically significant in univariate analysis or variables that did not show statistical significance in the current study but were reported to be risk factors in other literatures. Variables included in the logistic regression model were weight loss, diarrhea, well water for drinking, untreated drinking water, CD4^+^ < 200 cells/μL, and exposure to river water. Multivariate analysis showed that diarrhea (AOR 6.5; 95% CI 1.16–36.67), well water for drinking (AOR 6.7; 95% CI 1.83–24.93), and untreated drinking water (AOR 5.8; 95% CI 1.04–32.64) were risk factors for cryptosporidiosis in this study (Table 4).

## 4. Discussion

This study demonstrated a high prevalence of *Cryptosporidium* infection among children with HIV infection detected by molecular method. Previous studies reported the rates of cryptosporidiosis in the range 2.1% to 60.9% [7,8,9]. The occurrence of cryptosporidiosis might have been affected by study population, immune status, and diagnostic method [10]. In individuals with HIV infection, the prevalence of cryptosporidiosis was reported to be higher due to the suppression of cellular immune response causing higher risk for infection [10]. Among 474 malnourished children without HIV under 5 years of age, the prevalence of cryptosporidiosis was low (2.1%), with a higher proportion of infection reported in the food-weaning age group [7]. Another study in adults living with HIV resulted in a *Cryptosporidium* spp. infection rate of 60.9% [8]. However, the respected study involved only 32 subjects. These studies used stool microscopy and mZN staining as the diagnostic technique without PCR confirmation. The diagnostic method used in the studies might have also influenced the varying prevalence of cryptosporidiosis. Using PCR, the current study showed a cryptosporidiosis prevalence of 42.3%, while using microscopy, the prevalence was 38.5%. A pooled prevalence of cryptosporidiosis was reported to be higher using PCR (13.5%), followed by stool microscopy (10%) [1]. Molecular detection of *Cryptosporidium* in children living with HIV was performed in public hospitals in Kenya, resulted in a prevalence of 32% [27]. A wide range of diagnostic performance of microscopy compared to stool antigen detection and PCR has been reported in several studies [28,29]. Low infection intensity, intermittent oocyst shedding in mild or chronic infections, and morphological distortion of parasites might have caused lower microscopic detection in the current study. However, the possibility of false-negative PCR results should not be overlooked. The fecal samples might have contained PCR inhibitors including polysaccharides originating from a vegetable diet that might have caused false-negative results [30]. Therefore, interpretation should be made carefully depending on the study population, study size, disease severity, and diagnostic method used. 

Classic manifestations of intestinal cryptosporidiosis include non-bloody diarrhea, bloating, abdominal pain, and malaise [31]. Diarrhea is more commonly found to be persistent to chronic, causing symptoms of dehydration, malabsorption, and growth retardation [32]. In the current study, only 11 subjects reported diarrhea and 8 of them were found to be positive for *Cryptosporidium* spp. In immunosuppressed individuals, the clinical presentation of cryptosporidiosis can be atypical or even more severe [33]. Asymptomatic cryptosporidiosis has been reported in immunocompetent children [34] and in children living with HIV [35]. In our study, similar phenomena were seen, where 32% of the subjects with cryptosporidiosis did not experience any symptoms and diarrhea was observed more frequently among children with a higher HIV stage. A case report showed that subclinical cryptosporidiosis could be found in individuals with CD4^+^ < 100 cells/μL [36]. These asymptomatic individuals may serve as reservoirs for parasite transmission, thus endangering the environment [16]. Screening may be advisable in high-risk children, particularly those having CD4^+^ < 100 cells/μL and relevant risk factors, as this group may also present as having asymptomatic infections devoid of diarrhea [36]. 

Opportunistic infection is an important cause of morbidity and mortality in individuals with HIV infection [37]. The presence of opportunistic infection correlates to the severity of the HIV clinical stage [22]. In this study, 36% of the children with stage 1 HIV infection (most of them being on ART) were indeed found to have asymptomatic cryptosporidiosis, while those who experienced episodes of diarrhea belonged to HIV stages 3 and 4. In cryptosporidiosis, the principal immune response is cellular immunity mediated by the CD4^+^ cells that triggers IFN-γ production in response to *Cryptosporidium* spp. infection [38]. This may explain mild symptoms, or asymptomatic infection, in HIV individuals with higher CD4^+^ levels [38]. Opposing results have been reported in another study, showing that the risk of cryptosporidiosis in HIV individuals with CD4^+^ < 200 cells/μL did not differ with those above 200 cells/μL [39]. This might be due to variations in risk exposure to possible sources of infection and ART status [39]. Additionally, over 70% of the subjects enrolled in the study had only less than 5 years of ARV treatment, and many of these subjects did not visit the clinic due to travel restrictions during the early COVID-19 pandemic. This potentially affected the viral load as well as the CD4 levels of the subjects, hence their altered risks in getting opportunistic infections.

Multivariate analysis results in the current study showed the risk factors of cryptosporidiosis in HIV children as follows: diarrhea, well water for drinking, and untreated drinking water. The presence of diarrhea in children with HIV infection increased the odds of cryptosporidiosis by six times in this study. Similarly, other studies conducted in infants [40] and in school-age children [41] also showed that diarrhea, particularly persistent diarrhea, was more likely to be found in cryptosporidiosis. In younger children, *Cryptosporidium* spp. infection can lead to growth and developmental delay, primarily if the infection occurs within the first years of life [40]. 

The current study also showed that drinking well water and untreated water resulted in a higher risk of cryptosporidiosis of approximately 7- and 6-fold, respectively. Water contamination with *Cryptosporidium* spp. oocysts might have occurred through agricultural and animal runoff, and open defecation practices [42]. Boiling water for at least one minute was proven to be the most effective measure to deactivate *Cryptosporidium* spp. in drinking water [42]. However, in the current study, most of the subjects positive for cryptosporidiosis had boiled water for drinking in their daily life. The most likely cause of active *Cryptosporidium* despite water boiling was an improper practice of boiling water. Other sources of contamination may include contact with infected individuals, using contaminated water for washing fruits and vegetables, and consumption of raw fruits or vegetables. At most, these findings should increase awareness on the importance of drinking water quality, as well as proper health education in the household and community levels to prevent *Cryptosporidium* transmission. However, the source of infection and exact mode of transmission need further studies, such as *Cryptosporidium* species determination and detection of *Cryptosporidium* in the environment, which were not performed in this study.

This study had several limitations. Firstly, the study used a relatively small sample size in only outpatients, hence, prevalence should not be generalized to similar populations, and the results of statistical analysis should be interpreted carefully. Secondly, stool specimens were only collected once to detect the parasite, which might have impacted the detection rates. Lastly, sequencing was not performed to identify the *Cryptosporidium* species causing the infections in the study population. Nevertheless, there were significant findings in the current study that may contribute several discoveries in the respective field.

## 5. Conclusions

The current study highlights a high prevalence of asymptomatic cryptosporidiosis among mild-immunodeficient HIV children. Predictors of infection include a history of diarrhea, drinking well water, and drinking untreated water. Recent study findings should alarm attending clinicians to the burden of cryptosporidiosis as an AIDS-defining condition in children living with HIV. The study results should also increase awareness in governments and stakeholders of the importance of safe water supply, as well as encourage education for parents and caregivers towards proper food hygiene and sanitation practices.

## Figures and Tables

**Table 1:** General characteristics and clinical findings of study subjects.

Parameter		Total (N = 52)	*Cryptosporidium* spp. Infection					
			Negative (N = 30)	Positive (N = 22)	*p*-Value	Statistics Value ^a^	df	Effect Size ^b^
Mean age (years) ^†^		7.5 ± 4.0	8.1 ± 4.2	6.6 ± 3.5	0.174	1.379	50	0.388
Age group ^‡^								
	6–59 months	18 (34.6)	9 (30.0)	9 (40.9)	0.414 *	0.667	1	0.113
	5–12 years	26 (50.0)	15 (50.0)	11 (50.0)	>0.999 **	NA	NA	NA
	13–18 years	8 (15.4)	6 (20.0)	2 (9.1)	0.442 **	NA	NA	NA
Sex ^‡^								
	Female	21 (40.4)	13 (43.3)	8 (36.4)	0.613 *	0.256	1	0.070
	Male	31 (59.6)	17 (56.7)	14 (63.6)				
Comorbidities ^‡^								
	Tuberculosis	17 (32.7)	8 (26.7)	9 (40.9)	0.279 *	1.170	1	0.150
	Recurrent respiratory infections	6 (11.5)	5 (16.7)	1 (4.5)	0.226 **	NA	NA	NA
	Oral candidiasis	4 (7.7)	1 (3.3)	3 (13.6)	0.299 **	NA	NA	NA
	Other ^¶^	5 (9.6)	4 (13.3)	1 (4.5)	0.381 **	NA	NA	NA
	None	20 (38.5)	12 (40.0)	8 (36.4)	>0.999 *	0.071	1	0.037
Duration of HIV infection ^‡^								
	<1 year	9 (17.3)	3 (10.0)	6 (27.3)	0.144 **	NA	NA	NA
	1-5 years	29 (55.8)	18 (60.0)	11 (50.0)	0.473 *	0.515	1	0.099
	>5 years	14 (26.9)	9 (30.0)	5 (22.7)	0.559 *	0.341	1	0.081
ART status ^‡^								
	Treated	43 (82.7)	27 (90.0)	16 (72.7)	0.144 **	NA	NA	NA
	Untreated	9 (17.3)	3 (10.0)	6 (27.3)				
CD4^+^ lymphocyte levels								
	CD4^+^ (%) ^†^	21.8 ± 7.8	23.2 ± 8.2	19.9 ± 7.1	0.140	1.499	50	0.430
	CD4^+^ absolute (cells/μL) ^†^	751 ± 456	846 ± 517	622 ± 324	0.062	1.787	50	0.519
HIV clinical stadium ^‡^								
	Stage 1	20 (38.5)	12 (40.0)	8 (36.4)	0.790 *	0.071	1	0.037
	Stage 2	10 (19.2)	6 (20.0)	4 (18.2)	>0.999 **	NA	NA	NA
	Stage 3	17 (32.7)	8 (26.7)	9 (40.9)	0.279 *	1.170	1	
	Stage 4	5 (9.6)	4 (13.3)	1 (4.5)	0.381 **	NA	NA	NA
Diarrhea								
	No	41 (78.8)	27 (90.0)	14 (63.6)	0.037 **	NA	NA	NA
	Yes	11 (21.2)	3 (10.0)	8 (36.4)				
Nausea and/or vomiting								
	No	46 (88.5)	28 (93.3)	18 (81.8)	0.382 **	NA	NA	NA
	Yes	6 (11.5)	2 (6.7)	4 (18.2)				
Decreased appetite								
	No	42 (80.8)	24 (80.0)	18 (81.8)	>0.999 **	NA	NA	NA
	Yes	10 (19.2)	6 (20.0)	4 (18.2)				
Weight loss								
	No	33 (63.5)	15 (50.0)	18 (81.8)	0.019 *	5.542	1	0.326
	Yes	19 (36.5)	15 (50.0)	4 (18.2)				
Fever								
	No	49 (94.2)	30 (100)	19 (86.4)	NA	NA	NA	NA
	Yes	3 (5.8)	0	3 (13.6)				
BMI (kg/m^2^) ^§^ (N = 34)		14.1 (11.3–20.7)	14.3 (11.3–19.5)	13.9 (12.9–20.7)	0.649	NA	NA	NA
Nutritional status ^‡^								
	Normal	33 (63.5)	17 (56.7)	16 (72.7)	0.235 *	1.412	1	0.165
	Underweight	19 (36.5)	13 (43.3)	6 (27.3)				
Stunting status ^‡^ (N = 18)								
	Normal	14 (77.8)	7 (77.8)	7 (77.8)	>0.999 **	NA	NA	NA
	Stunted	4 (22.2)	2 (22.2)	2 (22.2)				

HIV: human immunodeficiency virus; ART: antiretroviral therapy; BMI: body mass index; stunting status: for children under five years. ^¶^ other: lymphoma, meningitis, Kaposi sarcoma. ^‡^ presented in frequency (%). ^†^ presented in mean ± SD, unpaired *t*-test. ^§^ presented in median (min–max), Mann–Whitney U test. * chi-square test. ** Fisher’s exact test. ^a^ chi-square value or *t*-test value. ^b^ Cramer’s V (chi-square) or Cohen’s *d* (*t*-test).

**Table 2:** Distribution of *Cryptosporidium* spp. infection in relation to the stages of HIV.

Parameter ^‡^		HIV Stage 1 (N = 8)	HIV Stage 2 (N = 4)	HIV Stage 3 (N = 9)	HIV Stage 4 (N = 1)
Age group					
	<5 years	3 (38)	0 (100)	6 (67)	0 (0)
	5–12 years	5 (62)	3 (75)	3 (33)	0 (0)
	>12–18 years	0 (0)	1 (25)	0 (0)	1 (100)
Nutritional status					
	Normal	8 (100)	4 (100)	4 (44)	0 (0)
	Underweight	0 (0)	0 (0)	5 (56)	1 (100)
CD4^+^ lymphocyte levels					
	≥200 cells/μL	5 (62)	3 (75)	3 (33)	0 (0)
	<200 cells/μL	3 (38)	1 (25)	4 (44)	1 (100)
	<100 cells/μL	0 (0)	0 (0)	2 (23)	0 (0)
ART status					
	Under therapy	6 (75)	3 (75)	6 (67)	1 (100)
	Untreated	2 (25)	1 (25)	3 (33)	0 (0)
Diarrhea					
	No	7 (88)	3 (75)	4 (44)	0 (0)
	Yes	1 (12)	1 (25)	5 (56)	1 (100)
Infection intensity *					
	Mild (<2 oocysts)	6 (75)	4 (100)	5 (56)	0 (0)
	Moderate (2–10 oocysts)	2 (25)	0 (0)	2 (22)	0 (0)
	Severe (>10 oocysts)	0 (0)	0 (0)	2 (22)	1 (100)

ART: antiretroviral therapy. ^‡^ presented in frequency (%). * determined by the number of oocysts found in a single immersion field.

**Table 3:** Risk factors of *Cryptosporidium* infection in children living with HIV.

Parameter ^‡^		Total (N = 52)	*Cryptosporidium* spp.					
			Negative (N = 30)	Positive (N = 22)	*p*-Value	Chi-Square Value	df	Cramer’s V
Contact with diarrheic family member								
	No	39 (75.0)	23 (76.7)	16 (72.7)	0.746 *	0.105	1	0.045
	Yes	13 (25.0)	7 (23.3)	6 (27.3)				
Exposure to river water								
	No	40 (76.9)	23 (76.7)	17 (77.3)	0.959 *	0.003	1	0.007
	Yes	12 (23.1)	7 (23.3)	5 (22.7)				
Exposure to animals								
	No	20 (38.5)	13 (43.3)	7 (31.8)	0.399 *	0.711	1	0.117
	Yes	32 (61.5)	17 (56.7)	15 (68.2)				
Raw vegetable consumption								
	No	32 (61.5)	17 (56.7)	15 (68.2)	0.399 *	0.711	1	0.117
	Yes	20 (38.5)	13 (43.3)	7 (31.8)				
Handwashing prior to eating								
	Yes	42 (80.8)	25 (83.3)	17 (77.3)	0.725 *	0.300	1	0.076
	No	10 (19.2)	5 (16.7)	5 (22.7)				
Drinking water source								
	Well water	30 (57.6)	12 (40.0)	18 (81.8)	0.003 *	9.094	1	0.418
	Tap/treated water	11 (21.2)	11 (36.7)	0	NA	10.231	1	0.444
	Bottled water, rainwater	11 (21.2)	7 (23.3)	4 (18.2)	0.741 **	NA	NA	NA
Water treatment								
	Boiled	41 (78.8)	27 (90.0)	14 (63.6)	0.037 **	NA	NA	NA
	Not boiled	11 (21.2)	3 (10.0)	8 (36.4)				

^‡^ presented in frequency (%). * chi-square test. ** Fisher’s exact test.

**Table 4:** Analysis of risk factors of cryptosporidiosis in children living with HIV.

Variables	Univariate Analysis		Multivariate Analysis	
	*p*-Value *	COR (95% CI)	*p*-Value	AOR (95% CI)
CD4^+^ < 200 cells/μL	0.496	1.5 (0.45–5.26)	-	-
Weight loss	0.019	2.6 (1.03–6.53)	-	-
Diarrhea	0.021	5.1 (1.18–22.49)	0.033	6.5 (1.16–36.67)
Exposure to river water	0.959	1.1 (0.28–3.82)	-	-
Well water for drinking	0.003	6.7 (1.82–24.93)	0.004	6.7 (1.83–24.93)
Untreated drinking water	0.021	5.1 (1.18–22.49)	0.045	5.8 (1.04–32.64)

COR: crude odds ratio; AOR: adjusted odds ratio. * chi-square test.

## Data Availability

The data presented in this study are contained within the article.

## Supplementary Materials

The following supporting information can be downloaded at https://www.mdpi.com/article/10.3390/tropicalmed7110352/s1. Table S1: Risk Behavior of Cryptosporidiosis-Positive Children Living With HIV. In Contrast to Drinking Water Treatment.
