# Supplementary material for: Asymptomatic Cryptosporidiosis in Children Living with HIV
Source: Trop Med Infect Dis. 2022 Nov 4;7(11):352. doi: 10.3390/tropicalmed7110352 (PMC9695426; doi:10.3390/tropicalmed7110352)
Supplement: Supplementary file 1 [file tropicalmed-07-00352-s001.zip › tropicalmed-1979937-supplementary.pdf]

Supplementary File

Table S1. Risk Behavior of Cryptosporidiosis-Positive Children Living With HIV. In Contrast to Drinking Water Treatment.

**Parameter**[‡]	**Total (N= 22)**	**Boiled (N = 14)**	**Not boiled (N = 8)**
Diarrhea			
No	14 (63.6)	9 (64.3)	5 (62.5)
Yes	8 (36.4)	5 (35.7)	3 (37.5)
Exposure to river water			
No	17 (77.3)	11 (78.6)	6 (75.0)
Yes	5 (22.7)	3 (21.4)	2 (25.0)
Contact with diarrheic family member			
No	16 (72.7)	10 (71.4)	6 (75.0)
Yes	6 (27.3)	4 (28.6)	2 (25.0)
Contact with animals			
No	7 (31.8)	5 (35.7)	2 (25.0)
Yes	15 (68.2)	9 (64.3)	6 (75.0)
Raw vegetable consumption			
No	15 (68.2)	10 (71.4)	5 (62.5)
Yes	7 (31.8)	4 (28.6)	3 (37.5)
Well water for drinking			
Yes	18 (81.8)	14 (100)	4 (50.0)
No	4 (18.2)	0	4 (50.0)

[‡]presented in frequency (%)
